# Extrinsic and intrinsic determinants of nerve regeneration

**DOI:** 10.1177/2041731411418392

**Published:** 2011-09-13

**Authors:** Toby A. Ferguson, Young-Jin Son

**Affiliations:** 1 Shriners Hospitals Pediatric Research Center, Temple University School of Medicine, Philadelphia, PA 19140, USA; 2 Department of Neurology, Temple University School of Medicine, Philadelphia, PA 19140, USA; 3 Department of Anatomy and Cell Biology, Temple University School of Medicine, Philadelphia, PA 19140, USA

**Keywords:** regeneration, spinal cord injury, sprouting, transplants, neurotrophic factor, PTEN, SOCS3

## Abstract

After central nervous system (CNS) injury axons fail to regenerate often leading to persistent neurologic deficit although injured peripheral nervous system (PNS) axons mount a robust regenerative response that may lead to functional recovery. Some of the failures of CNS regeneration arise from the many glial-based inhibitory molecules found in the injured CNS, whereas the intrinsic regenerative potential of some CNS neurons is actively curtailed during CNS maturation and limited after injury. In this review, the molecular basis for extrinsic and intrinsic modulation of axon regeneration within the nervous system is evaluated. A more complete understanding of the factors limiting axonal regeneration will provide a rational basis, which is used to develop improved treatments for nervous system injury.

## Introduction

Understanding the mechanisms of axonal regeneration and improving human recovery after nervous system injury has been a long-standing goal of the neuroscience and medical community at least since the time of Cajal if not longer. Injured peripheral axons mount a robust regenerative response, and functional recovery in humans is possible, although such recovery is often limited by the long distance that regenerating peripheral axons must grow. After central nervous system (CNS) injury, axonal regeneration is less successful and often only limited recovery is possible. In a series of now famous nerve transplantation studies, David and Aguayo demonstrated that peripheral nerve grafts support robust growth of some central axon populations.^1^ The initial understanding of these experiments emphasized the growth-inhibitory nature of the CNS environment, and subsequent work has elucidated multiple myelin and proteoglycan growth-inhibiting components found in the CNS.^2^ However, attempts to improve axonal regeneration and functional recovery by ablating these inhibitory molecules by either pharmacologic or genetic manipulations have met with only minimal (proteoglycan-based inhibitors) or no success (myelin-based inhibitors).^3^–^5^ Therefore, a modern interpretation of Aguayo and Bray’s famous experiments would suggest that the intrinsic growth state varies among neuronal populations and likely determines the ability of a neuron to either overcome an inhibitory environment or avail themselves of a growth-permissive environment. Indeed, removal of general, growth-suppressing genes notably improves regeneration of neuronal populations refractory to regeneration.^6^

Therefore, in this review we will discuss critical factors affecting the ability of injured peripheral and central neurons to generate an effective growth response. We will emphasize recent findings that suggest the possibility of modulating a neuron’s growth response to injury.

## Molecular signaling of axon injury

### Calcium

In order to generate a successful response to injury, injured axons must first detect axonal damage. Local axonal damage triggers both rapid calcium-based and slower retrograde transport-based signals.

Extracellular calcium enters a damaged axon, elevates axoplasmic calcium concentration, and may trigger rapid growth cone formation in *Aplysia*.^7^ Acute axotomy of cultured mammalian neurons also triggers an increase in calcium concentration in the distal tip of the damaged axon. Subsequently, a calcium wave is retrogradely propagated to the soma by voltage-dependent sodium channel activation of a transient calcium current.^8^ Calcium transients also correlate with regenerative growth and this growth depends, in part, on a dual leucine kinase-1 (DLK-1), a conserved member of the mitogen-activated protein kinase (MAPK) pathway, in *Caenorhabditis elegans*.^9^ This kinase has also been found to be required for growth cone formation and regeneration throughout the *C. elegans* life cycle^10^ and in drosophila.^11^ In mice, depletion of DLK-1 sensory neuron outgrowth in vitro and phosphorylation of *c-jun* in vivo are reduced.^12^ The effect of DLK-1 may be mediated by enhancement of CCAAT/enhancer-binding protein-1 (C/EBP-1) mRNA stability.^13^ Calcium, therefore, quickly signals acute axonal injury and, in part, initiates a regenerative response through DLK-1.

### Retrograde signals of axonal injury

In addition to the calcium-generated injury response, slower, retrogradely transported proteins also signal axonal injury to the cell body. Best characterized of these molecules are the importins. After injury, local axonal synthesis of β1-importin allows assembly of a macromolecular complex and retrograde transport of nuclear localization sequence (NLS)-containing cargo to the nucleus of sensory neurons. Excess exogenous NLS peptide slows regenerative growth.^14^ Multiple importin-binding cargos have been identified, but in neurons, translocation of vimentin-bound MAP kinase extracellular signal-regulated kinase (ERK)^15^ and the transcription factor CREB2^16^ clearly suggest retrograde transported cargo signal injury to the cell body. At least in sensory neurons, the initiation and loading of cargo appear dependent on axonal Ran-binding protein 1 (RanBP1).^17^ Other possible signals include Smads, which are also regulated after peripheral nerve injury.^18,19^ At least one, Smad1, appears important for initiation or maintenance of sensory neurite outgrowth.^18^ Wallenda, the drosophila homologue of DLK, is required for injury signaling and is regulated by an E3 ubiquitin ligase highwire.^11^ Retrograde transport of a c-Jun NH2-terminal kinase (JNK) scaffolding protein, Sunday Driver,^20^ multiple JNK signaling molecules, and ATF3 have also been observed.^21^ Given the observed variable growth competence of CNS and peripheral nervous system (PNS) neurons to regenerate, it is possible that neuronal populations signal damage with differing efficacy. Nonetheless, it is clear that retrograde signals participate in signaling axonal damage and aid initiation of a growth response.

## Neuronal cell death and regeneration

In order to regenerate, a neuron must survive the severing of its axon. During development, axotomy has been clearly linked to cell death. However, in the adult CNS, survival and regeneration appear distinct. After optic nerve axotomy, many retinal ganglion neurons survive axotomy if a peripheral nerve graft is placed near the neurons. However, only a small number of these surviving neurons regenerate into the graft.^22^ In addition, overexpression of *bcl-2*, an antiapoptotic gene, in retinal ganglion cells (RGCs) improves survival but does not substantially increase axon growth into a peripheral nerve graft.^23^ Thus, although neuronal survival is a necessary prerequisite for regeneration, survival itself does not confer a neuron with the ability to regenerate.

## Extrinsic modulation of the neuronal growth response

The intrinsic growth capacity of an injured neuron is largely influenced by the external environment. Some of the important extrinsic signals are presented in the following sections, and intrinsic drivers of regeneration are considered next.

### Cytokines

After axonal injury in vivo, the inflammatory environment contributes substantially to the neuronal injury response. Local inflammation near central or peripheral neuronal cell bodies elicits improved axonal growth, as first demonstrated by injecting either *Cornyebacterium* or activated macrophages into lumbar sensory neurons and measuring axonal regeneration after dorsal root crush.^24^ Macrophages also appear to play a similar key role in augmentation of CNS axonal growth. After injury of the optic nerve, macrophage-derived oncomodulin produced following the injection of zymosan or after lens injury promotes RGC regeneration.^25^–^27^ In addition, ciliary neurotrophic factor (CNTF) and leukemia inhibitory factor (LIF) are likely important in the retinal ganglion cell injury response as CNTF and LIF null animals failed to regenerate optic nerve axons after combined optic nerve crush and lens injury.^28^ Despite this observation, purified, exogenous cytokines only moderately improve CNS regeneration.^29,30^ Recent important experiments have provided a possible explanation for these observations. In particular, suppressor of cytokine signaling (SOCS) proteins may importantly limit the effectiveness of endogenous and exogenous cytokines in stimulating CNS regeneration. SOCS proteins are cytoplasmic inhibitors of JAK-STAT signaling.^31,32^ Regeneration was markedly improved using viral-mediated delivery of Cre (Cre recombinase) to the retina of SOCS3^fl/fl^ mice prior to optic nerve crush.^33^ The successful regeneration correlated with a reversal of mTOR pathway activity. Concurrent deletion of gp130 and SOCS3 also abrogated successful regeneration, suggesting that cytokine-mediated gp130 activation is required. Furthermore, STAT3 phosphorylation was severely decreased in these animals. Finally, viral-mediated overexpression of SOCS3 abrogates optic axon regeneration into a peripheral nerve graft.^34^ Together these observations suggest that CNTF and LIF may successfully initiate axonal regeneration in RGCs if the JAK-STAT signaling cascade is suitably activated. Likewise in sensory neurons, SOCS3 overexpression inhibits neurite outgrowth, at least in part, through inhibition of STAT3 signaling.^35^ Thus, while inflammation may clearly promote regeneration under certain circumstances, this response is also curtailed by neuronal expression of SOCS proteins, and in certain CNS neurons, this suppression may be powerful enough to limit regenerative growth.

### Neurotrophins

Neurotrophins and neurotrophic factors critically regulate the developmental cell survival and differentiation. In vitro, exogenous neurotrophin application increases axon growth of multiple neuron types.^36^ However, the in vivo role of individual neurotrophins in regeneration is less clear and likely complex. After peripheral nerve injury, Schwann cell production of multiple neurotrophins dramatically increases and is thought to be important for regeneration.^37^ Importantly, elevated levels of neurotrophins do not persist in chronic axotomy.^38^ In agreement with this observation, addition of exogenous brain-derived neurotrophic factor (BDNF) or glial-cell derived neurotrophic factor (GDNF) after acute or chronic sciatic nerve injury increased axon sprout formation at the injury site but did not increase the number of successfully regenerated motor neurons.^39,40^ However, exogenous administration of either BDNF or GDNF after chronic injury did improve motor axon regeneration.^39,40^ Therefore, neurotrophin-dependent axon sprout formation after injury may not necessarily equate to successful regeneration and only in certain circumstances are BDNF and GDNF important for peripheral axonal regeneration.

In multiple studies, exogenous neurotrophins delivered to the injured CNS have demonstrated increased axon growth.^41^ However, in these studies, either regeneration of axons has been modest^42^ or distinguishing long-distance axonal regeneration from sprouting of uninjured axons has been difficult. Most impressive have been studies of neurotrophin application after dorsal root crush. After crush injury, dorsal root axons regenerate within the peripheral nerve root but stall shortly after entering the CNS, perhaps because of rapid presynaptic differentiation at the CNS/PNS border.^43^ However, on intrathecal application of nerve growth factor (NGF), neurotrophin-3 (NT-3), or GDNF, dorsal root axons reinnervated the dorsal horn and partially reestablished sensory function.^44^ Similarly, systemic artemin not only allowed dorsal root ganglion (DRG) axon growth across the dorsal root entry zone but also restored both nociceptive and sensorimotor function.^45^ Furthermore, after artemin administration (but not Nogo receptor blockade), DRG axons regenerated to topographically appropriate region of the spinal cord dorsal horn.^46^ BDNF was not effective in this model. These observations suggest exogenous neurotrophins may be useful in promoting functional recovery after injury of DRG central processes. However, addition of exogenous neurotrophins does not define a biologic role for these molecules, which is likely complex and dependent on cell type. For example, cell-specific deletion of TrkB, the cognate BDNF receptor, from retina demonstrated that a model of toxic injury loss of TrkB from glial cells was as deleterious as loss of TrkB from neurons. Therefore, TrkB signaling in glial cells may be as important for RGC protection as neuronal TrkB signaling. Furthermore, loss of TrkB signaling in glial cells impaired glial proliferation and dedifferentiation after toxic insult.^47^ Clearly, defining both the biologic and possible therapeutic roles for neurotrophins in regeneration will require carefully designed experiments that address the functions of neurotrophins and their receptors within both neurons and glial cells.

### Glia-associated growth inhibitors

Experiments of the last two decades have well characterized exogenous neuronal growth inhibitors and recent reviews exist.^2^ Of these, the best described extrinsic growth-inhibitory molecules include the following: myelin-associated glycoprotein (MAG),^48,49^ Nogo,^50^–^52^ and oligodendrocyte-myelin glycoprotein (OMgp).^53,54^ These molecules are synthesized by oligodendrocytes and distributed in the myelin ensheathing CNS axons. All three myelin inhibitors bind to the glycosylphosphatidylinositol-anchored Nogo-66 receptor (NgR1), which is expressed by many CNS neurons.^54^–^56^ NgR1 antagonist treatment enhanced neurite outgrowth from DRG cells in co-culture model. Other receptors have also been implicated in mediating the inhibitory effect, including NgR2 and the paired immunoglobulin-like receptor B (PirB).^57,58^

The other major group molecules that inhibit neural regeneration are the chondroitin sulfate proteoglycan (CSPG) inhibitors. Chondroitin 6-sulfate proteoglycans are produced by astrocytes and associated with glial scar, which plays a major role in the regenerative failure after CNS injury.^59^–^61^ Neuroglycan 2 (NG2), aggrecan, brevican, neurocan, vesicant, and phosphacan are all different members of CSPG family of extracellular matrix molecules.^62^ The inhibitory property of CSPG has been attributed to its glycosaminoglycan (GAG) side chains, and enzymatic removal of GAG chains by chondroitinase ABC (ChABC) has been shown to promote axon regeneration both in vitro and in vivo.^3,63^–^66^ Recently, a transmembrane protein tyrosine phosphatase, PTPσ, was identified as a high-affinity receptor of CSPG, which mediates its inhibitory effect.^67^ Despite convincing in vitro data on the role of these growth-inhibitory molecules, knockout of the three myelin inhibitory proteins,^4^ or the NgR,^68^ has not improved corticospinal tract axonal regeneration. Enzymatic removal of CSPG or genetic deletion of PTPσ^3,69^ modestly improved axonal growth, although it is unclear if the observed growth is long-tract axonal regeneration or sprouting of spared axons. Thus, while the myelin and proteoglycan inhibitory molecules play some role in limiting axonal growth, additional limitations on axonal regenerative growth likely exist.

## Intrinsic modulation of neuronal growth response

### Endogenous growth suppressors

Discouraged by the poor regeneration observed after removal of myelin-based extrinsic growth inhibitors, investigators have attempted to define endogenous inhibitors of axonal regeneration. Early successes have focused on the molecule phosphatase and tensin homologue (PTEN) deleted on chromosome 10. Originally discovered as a tumor suppressor,^7^ PTEN deletion has been recently shown to dramatically increase postembryonic regeneration after injury in RGCs and corticospinal neurons in the CNS^70,71^ and axon outgrowth in the PNS.^72^ Loss of PTEN likely results in phosphatidylinositol (3,4,5)-trisphosphate (PIP_3_) accumulation, deregulation of AKT signaling, and likely increases regeneration through multiple downstream signaling effectors (for recent review see Ref. 6). Of particular note, administration of rapamycin (a known inhibitor of the mTOR pathway) significantly impaired PTEN deletion–mediated regeneration^6^ and, additionally, deletion of the endogenous mTOR inhibitor; tuberous sclerosis complex-1 (TSC1) also increased regeneration after optic nerve crush.^70^ These important observations suggest that the impressive CNS regeneration observed in these experiments depends, at least in part, on the ability of the neuron to initiate new protein synthesis needed to manufacture the raw material required for axonal regeneration. However, TSC-deleted regeneration was not as robust as PTEN-mediated regeneration suggesting some importance of signaling not dependent on new protein synthesis. Despite the substantial growth observed with PTEN deletion, regenerating axons did not reach the lateral geniculate. At least in RGCs, true target reinnervation will likely only result with combinatorial treatments including PTEN deletion, activation of inflammation, and elevation of cyclic adenosine monophosphate (cAMP).^73^

Also downstream from PTEN but likely independent of the mTOR pathway, glycogen synthesis kinase (GSK) also importantly regulates axon growth. Pharmacological PTEN inhibition increases phospho-AKT (pAKT) and pGSK-3β levels and neurite outgrowth suggesting one effector of PTEN inhibition–mediated outgrowth is GSK-3β.^74^ GSK-3 integrates multiple extracellular signals to modulate axon formation and elongation both at the cell body and growth cone.^6^ Neurotrophin-dependent inactivation of GSK-3β increases adenomatous polyposis coli (APC)^75^ or collapsin response mediator protein-2 (CRMP-2)^76^ stabilization of microtubules and increases axon elongation in developing neurites. Interestingly, basal GSK-3β activity likely prevents ongoing axon formation.^77^ More recent data suggest that GSK-3β likely plays a similar role in adult neurons^78,79^ and pharmacologic inhibition may improve raphespinal and corticospinal growth after spinal cord injury.^80^ However, GSK-3β may also respond to myelin inhibitors and, therefore, may regulate growth in a more complex fashion.^81^

### Cyclic nucleotides

Loss of neuronal cAMP, in part, accounts for developmental loss of regenerative ability,^82^ and spinal cord injury results in reduction of neuronal cAMP levels.^83^ Raising cAMP levels in a poorly regenerating zebrafish axons improved regeneration.^84^ Exogenous db-cAMP injection into dorsal root ganglia improves axonal growth into a spinal cord lesion,^85^ and local injection of a phosphodiesterase inhibitor (which elevates cAMP levels) improves axonal regeneration and functional recovery after spinal cord injury^86^ or regeneration after peripheral nerve injury.^87^ It is unclear if cAMP’s salutary effect on in vivo axon growth occurs at the axon tip, cell body, or both. Certainly, the effects of growth inhibitors at the growth cone are dependent on the neuronal levels of cAMP. When levels are high, the effect on the growth cone is chemoattraction, whereas when they are low, the effect is chemorepulsion,^88^–^90^ but the importance of these observations in regeneration is not clear. Interestingly, regeneration of spinal axons of lamprey is accelerated by exogenous cAMP, even though these axons do not appear to have classic growth cones.^91^

In regenerating zebrafish axons, cAMP dependent–regeneration required DLK-1 kinase.^9^ Moreover, cAMP modulates expression of SOCS molecules in the retina, which are known to limit cytokine-induced growth, and may provide an additional mechanism by which cyclic nucleotides augment regeneration.^92^ These studies suggest that cAMP prepares the neuron for a regenerative response.

### Transcription factors

In response to injury, some neurons dramatically alter their patterns of gene expression and switch to a regenerative phenotype, whereas other neuronal populations do not substantially alter their gene expression or abort an early regenerative response and thus fail to regenerate an axon. The coordinate regulation of these responses is likely controlled by multiple transcription factors and recent experiments have begun to elucidate both the basis of a successful regenerative phenotype after injury and the concomitant loss of regenerative ability that often occurs developmentally in CNS axons.

A study of developmentally regulated RGC genes revealed that multiple members of the Krüppel-like factors (KLFs) are present and developmentally regulated in RGCs and, therefore, putative regulators of axon growth.^93^ KLFs are a family of zinc-finger-containing transcription factors that regulate diverse biological processes.^94^ KLF-4 and KLF-9 expression increase at birth but KLF-6 and KLF-7 decrease, suggesting that these molecules may control the growth potential of retinal ganglion neurons. Indeed, deletion of KLF-4 improves neurite growth in vitro and optic nerve regeneration after crush in vivo.^93^ In addition, optic axons successfully regenerate in adult zebrafish and increase their expression of KLF6a and KLF7a after injury. Furthermore, simultaneous knockdown of both these molecules impaired RGC outgrowth.^95^ Interestingly, KLF knockdown impaired regeneration-dependent expression of alpha 1 tubulin, which is likely important for axon extension.^96^ These observations demonstrate that the regenerative competence of neurons is actively regulated by groups of functionally interrelated transcription factors but during development and after injury. Furthermore, manipulation of these factors can improve neuronal regenerative potential.

However, it is likely that the complex control of the regenerative response is an ‘all or none’ phenomenon, but instead discrete aspects of regeneration are likely controlled by separate transcription factors. STAT3, as part of the JAK-STAT signaling pathway, is activated after peripheral but not central axon lesion^97^ and pharmaceutical blockade of STAT3 activation after peripheral nerve injury prevents CNS axon growth normally observed after a conditioning lesion.^98^ These observations suggest that STAT3 plays a critical role in the neuronal response to injury. However, recent experiments have importantly refined the role of STAT3 in regeneration. After selective deletion of sensory neuron STAT3, the initiation of peripheral regeneration after nerve transection stalled but subsequent axonal elongation was unaffected. In addition, STAT3 overexpression in DRGs increased sprout formation after dorsal column lesion but did not lead to persistent axonal growth.^99^ Finally, STAT3 overexpression also increased neurite outgrowth of cerebellar granule cells in vitro.^100^ Therefore, STAT3 importantly regulates the initiation but not continuation of axonal regeneration in both central and peripheral axons. In general, these observations suggest that individual signaling pathways and downstream transcription factors may control discrete phases of the regenerative process.

Interestingly, some transcription factors increase after injury but appear to limit the regeneration response. Nuclear factor IL-3 (NFIL3) regulation increases in sensory neurons after injury but represses CREB-mediated transcription and expression of regeneration-associated genes such as arginase and GAP-43, thereby likely limiting the regenerative response.^101^ More recently, NFIL3 has been shown to also repress expression of genes activated by C/EBP family members.^102^ Therefore, even successfully regenerating neurons may endogenously limit their growth response.

Other notable transcription factors include members of the Jun and Fos families, components of the transcription factor AP-1 and ATF3. Cre-mediated *c-jun* deletion in the CNS impaired regeneration of facial motor axons, abrogated upregulation of several other regeneration- associated molecules, and impaired microglial activation.^103^ ATF3 increases after peripheral nerve axotomy in motor and sensory neurons.^104^ Expression may also be seen in corticospinal neurons with intracortical but not spinal cord lesions,^105^ thalamic nuclei that have grown into a peripheral nerve graft,^106^ or zebrafish retina, which can successfully regenerate axons.^107^ Transgenic overexpression of ATF3 in dorsal root ganglia increased the regenerative capacity of peripheral DRG axons suggesting ATF3 expression may help determine the regenerative state of sensory neurons.^108^

## Effectors of axonal regeneration

After injury in the CNS, cut axons may attempt to regenerate. However, despite these attempts, damaged axons do not regenerate to significant distances. Axonal growth failure is a result of both the neuron’s poor intrinsic growth potential and the growth-inhibitory environment of the injured CNS. Recent studies of intrinsic growth regulators have emphasized the importance of new protein synthesis and axonal transport as critical determinants of regeneration, and other studies have emphasized regeneration-associated molecules that correlate with successful regeneration. Therefore, the many growth-associated molecules that are regulated by intrinsic determinants of regeneration likely play a critical and specific role in the regenerative process and some are highlighted in the following sections.

### Conditioning lesions

If a cut regenerating axon is reinjured more proximally, it will grow at a greater rate than if the axon suffered only one injury. This is the classic conditioning lesion.^109^ Most remarkably, sciatic nerve transection prior to a dorsal column lesion of the ascending sensory neuron projections remarkably improved regeneration within the injured CNS.^110^ Subsequent studies found a substantial increase in cAMP in injured DRG and that db-cAMP injections partially replicate the effect of a conditioning lesion.^85^ Interestingly, interleukin (IL)-6 injections also mimic this response but IL-6 is not required for a conditioning lesion effect.^111^ More recent experiments have found that cultured superior cervical ganglia neurons did not respond to conditioning lesion if gp130 was deleted.^112^ One downstream effect of a conditioning lesion is increased transport of tubulin and actin.^113^ However, in poorly regenerating RGCs, axonal transport of cytoskeleton proteins decreases 10-fold unless axons encounter and regenerate into a peripheral nerve grafts.^114,115^ Although not fully understood, the improved regeneration observed after conditioning lesion most importantly emphasizes the critical role that neuronal growth state plays in successful regeneration, even in the injured CNS and further suggests that associated differences in axonal transport may play a role in growth efficacy.

### Growth-associated proteins

After sciatic nerve transection, at least an estimated 240 genes in DRG undergo dramatic up or down regulation,^116^ including a number of transcription factors that undergo early change after injury.^117^ Presumably, this coordinated response regulates genes critical for conversion of a neuron to a growth-promoting phenotype and, therefore, successful axonal regeneration. Of these growth-associated genes, GAP-43 is best known, although its precise function is unclear.^118,119^ Cell type–specific overexpression of GAP-43 in transgenic mice did not induce axonal regeneration in these cells, although short-distance sprouting did occur. These findings can be interpreted to imply that GAP-43 is important in the generation of growth cones but this is not sufficient to induce regeneration. However, when GAP-43 and cytoskeleton-associated protein of 23 kDa (CAP-23) were overexpressed together, DRG axons were able to regenerate after spinal cord transection.^120^ In transgenic zebrafish, a GAP-43 promoter element that triggered expression of GAP-43 during axon development did not do so during regeneration of optic nerve.^121^ Thus, the signaling pathways for axon elongation during regeneration may be different from those during axon development.

### Cytoskeletal proteins

During development, growth cones are found at the growing tips of axons and consist of filopodia and lamellipodia. Embedded in these structures are cell surface receptors that translate surface binding into the intracellular signals that regulate axon elongation, turning, and growth inhibition. The role of cytoskeletal elements in adult neuron regeneration is less clear, although presumably important. During CNS regeneration in mammals, growth cones are poorly formed, with a bullet-like appearance and dystrophic axon retraction bulbs are commonly observed. During peripheral nerve regeneration, synthesis of tubulin and actin is increased but neurofilament is downregulated^122^ suggesting that microtubules and actin microfilaments are critical for regeneration. Importantly, in the CNS, only axons regenerating into a peripheral nerve graft express tubulin,^123^ suggesting that the CNS environment may limit the expression of cytoskeletal elements important for axon extension. In a morphologic analysis of regenerating axons, Ertuk and colleagues found that microtubules in retraction bulbs were not arrayed along the longitudinal axis of the axon in contrast to axons without retraction bulbs. Local administration of taxol, a microtubule-stabilizing agent, interfered with retraction bulb formation after dorsal column lesion and increased in vitro neurite outgrowth on myelin.^124^ After optic nerve crush, local taxol administration in combination with lens injury notably increased axon growth, perhaps through reduction of local CSPG production.^125^ Similarly, after peripheral conditioning and dorsal column lesions, sensory axons grew into glial scar after local taxol administration possibly because of decreased TGF-β1 signaling and CSPG production.^126^ Surprisingly, regenerative axon elongation does not appear to require a microtubule-organizing center.^127^ Thus, it may be that microtubules (MTs) assembled locally in a growing axon and are important for regeneration and responsive to local inhibitory molecules.

### Cell adhesion molecules

In the CNS and PNS, regenerating axons often grow on cell surfaces.^71,128^–^130^ In the PNS, cell adhesion molecules embedded in the Schwann cell membrane are thought to contribute to the success of regeneration, whereas in the injured CNS, the cellular environment and their respective cell adhesion molecules are a complex mix of growth-inhibitory and growth-promoting molecules, which, on balance, are thought to impede regeneration.^2,130^ The molecules of the Schwann cell membrane are thought to facilitate axonal regeneration that includes neuronal cadherin (NCAD) and L1, among others.^131^–^134^ NCAD, L1, and neuronal cell adhesion molecule (NCAM) are thought to be important for embryonic neurite growth on astrocytes in the CNS.^135^–^137^ After injury, adult PNS neurons and glia increase expression of these growth-associated cell adhesion molecules,^138^–^140^ but in the CNS, poorly regenerating neurons such as RGCs or corticospinal neurons do not reexpress these molecules after injury.^141,142^ In zebrafish, the regenerative abilities of different neurons correlated with expression of the homophilic cell adhesion molecule L1 and NCAD.^143,144^ Furthermore, L1 knockdown in motor neurons impaired axonal regeneration.^145^ Thus, adhesion molecules are able to help overcome an inhibitory environment and tip the balance to favor axon regeneration. Following this logic, forced expression of cell adhesion molecules improves CNS recovery after injury. For example, increased L1 or L1 and GAP-43 expression improved Purkinje cell regeneration into a peripheral nerve graft,^146^ and virally mediated L1 expression at the site of spinal cord injury stabilized the corticospinal tract, enhanced the growth of 5-HT axons, and correlated with moderate functional improvement.^147^ In conclusion, earlier reports demonstrated that after spinal cord injury in adult rats, treatment with soluble L1-Fc promotes axon regeneration and functional recovery.^148^ Therefore, the ability of neurons to express complimentary cell adhesion molecules on their surface is likely one important intrinsic determinant of their regenerative ability.

## Summary and conclusions

The injured human CNS has only limited ability to recover after injury and little of this recovery appears to correlate with true, long-distance axonal regeneration. Studies carefully delineating the multiplicity of glial-inhibitory molecules and their axonal receptors generated widespread enthusiasm that removal of these molecules would improve both recovery and long-distance axonal regeneration. However, such removal does not appear to have had a dramatic effect on either functional recovery or long-distance axonal regeneration. More recent experiments suggest that robust long-distance regeneration potential declines developmentally and may be further suppressed after injury. Strikingly, removal of an array of growth suppressor molecules allows robust, long-distance axonal growth, presumptively by activating a regenerative response not normally accessible to most injured CNS neurons. These observations again provide hope that rational manipulation of the injured CNS will provide successful treatments after CNS injury. However, given the inherent glial and neuronal complexity of the injured CNS, it is unlikely that a single treatment approach will repair the CNS after injury. Instead, combination treatments will be increasingly prominent and indeed have shown promise experimentally.^73^
